# Infantile sternal osteomyelitis: A case of ultrasound tracking of bone displacement and regeneration

**DOI:** 10.1111/ped.70150

**Published:** 2025-08-28

**Authors:** Hiroki Shimono, Akiko Kinumaki, Natsuho Adachi, Hiroyuki Tanaka, Yuko Kajiho, Shoichiro Kanda

**Affiliations:** ^1^ Department of Pediatrics The University of Tokyo Hospital Tokyo Japan

**Keywords:** bone displacement, infant, new bone formation, sternal osteomyelitis

Primary sternal osteomyelitis is a rare infection of the bones, but its fundamental characteristics mirror those of long bone osteomyelitis: increased incidence at a younger age, male preponderance, nonspecific presentation, and unreliability of plain radiographs for diagnosis. Also, the spectrum of reported pathogens roughly agrees with that reported for osteomyelitis at other sites.^1^ Osteomyelitis complicated by abscesses or sequestra typically requires surgical intervention, such as abscess drainage or necrotic bone removal.^2^

A 1‐month‐old male infant developed a fever along with symptoms such as irritability and decreased milk intake and was admitted to another hospital. At the time of admission to the previous hospital, a viral infection was suspected, and no antibiotics were administered. However, a blood test on the following day showed an increase in C‐reactive peptide/protein (CRP; 7.14 mg/dL), leading to the initiation of ampicillin and cefotaxime. On the third day of hospitalization in the previous hospital, the blood culture was found to be positive. On the fourth day, methicillin‐susceptible *Staphylococcus aureus* (MSSA) was identified in the blood culture, and based on the susceptibility test results, antibiotics were switched to cefazolin. However, due to a temporary relapse of fever on the fifth day of hospitalization, the antimicrobial agent was switched back to cefotaxime. The blood culture obtained 4 days after initiating antimicrobial therapy yielded negative results. Subsequently, redness and swelling appeared in the anterior chest area. Chest magnetic resonance imaging (MRI) and computed tomography (CT; Figure 1a) indicated an anterior chest abscess. The patient was transferred to our hospital 10 days after admission to the previous hospital for potential surgical intervention. On admission to our hospital, ultrasonography revealed multiple sternal segments deviated towards the mediastinum, with a soft hypoechoic area in the sternum's original position. We considered osteomyelitis with abscess formation or a tumorous lesion causing bone destruction. We have changed antibiotics to cefazolin and continued antibiotic treatment, which led to the shrinkage of the hypoechoic lesion on ultrasonography (Figure 1b,c). The clinical course and examination findings strongly suggested sternal osteomyelitis with abscess formation and separated bone fragments. Although we considered surgical interventions such as drainage or removal of the detached bone fragments, we opted to continue antibiotic therapy without surgical intervention for the following reasons:

1. The causative organism was identified from blood culture.
2. The lesion had shrunk with antibiotic treatment, leaving no drainable cavity.
3. Surgical treatment of the posterior sternal lesion was deemed a highly invasive and high‐risk procedure.

**FIGURE 1:**
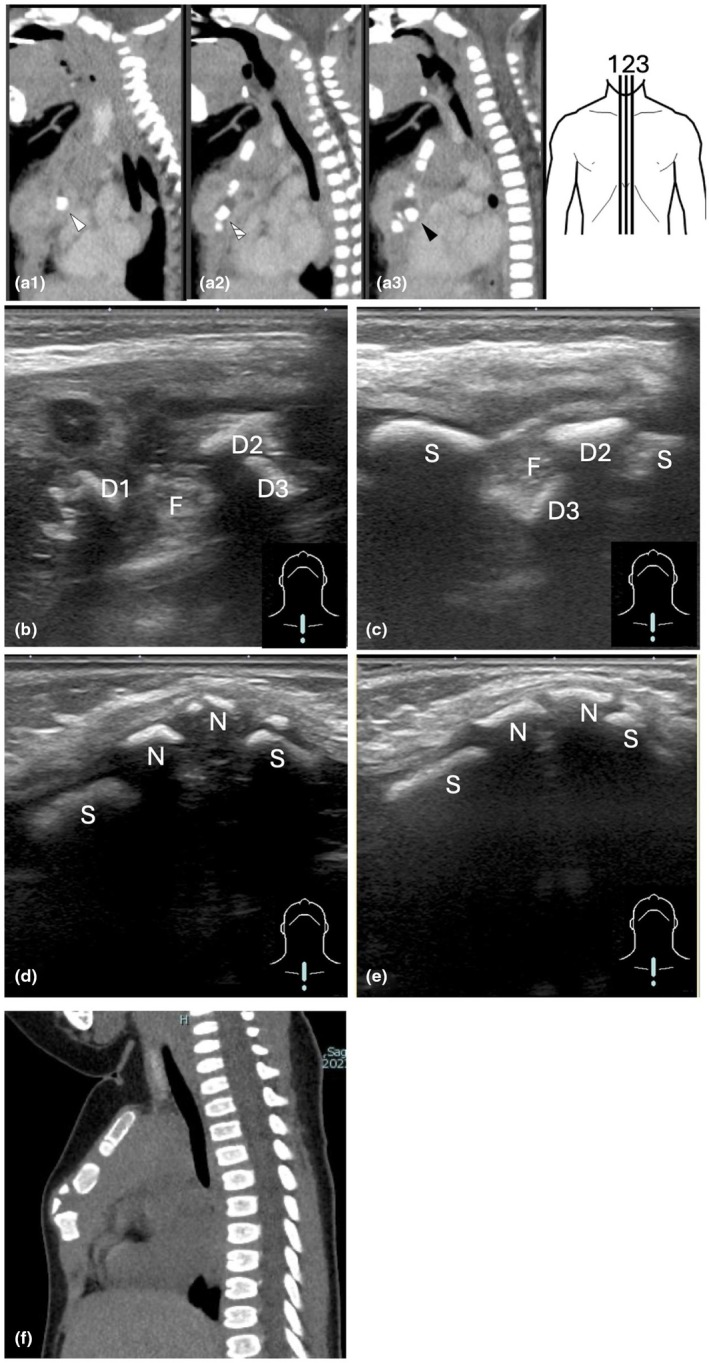
(a) CT image taken 1 day before admission to our hospital: Sternal segments are displaced posteriorly, with fluid accumulation in its original position. The fluid collection measured 15 mm in the transverse (left‐to‐right) direction, 12 mm in the craniocaudal (head‐to‐tail) direction, and 5 mm in the anteroposterior (front‐to‐back) direction. a1, a2, and a3 correspond to the CT slices at positions 1, 2, and 3 in the schematic diagram, respectively. (a1) Slightly right of the patient's midline: A completely displaced segment (white arrowhead) is visible in the mediastinum. (a2) At the patient's midline: A displaced segment (hatched arrowhead) is seen within the fluid‐filled cavity. (a3) Slightly left of the patient's midline: A segment (black arrowhead) moved posterior to the fluid‐filled cavity (F). (b) Ultrasonography image 2 days after admission: The fluid‐filled cavity (F) and displaced bone fragments (D1, D2, D3) confirmed by CT are visible. The left side of the image corresponds to the patient's cranial end. D1, D2, and D3 correspond to the bone fragments visible in images a1, a2, and a3, respectively. (c) Ultrasonography image at 14 days after admission: The fluid‐filled cavity between the original sternal segments (S) decreased in size compared to that at 2 days after admission. The displaced bone fragment D1 became less distinct. (d) Ultrasonography image at 2 months after discharge: The displaced segments are no longer visible. New segments (N) appeared in the sternum's original position. (e) Ultrasonography image at 3 months after discharge: The alignment of the newly appeared segments improved at 3 months compared to the previous month. (f) CT image at 6 months after discharge: Consistent with the ultrasonography findings, new segments can be observed in the sternum's original position. No residual fluid‐filled cavities or displaced segments are seen in other slices.

The patient completed 4 weeks of intravenous antibiotic therapy, which was switched to oral cefalexin and then he was discharged. Regular outpatient ultrasonography confirmed the disappearance of the detached sternal segments and new bone formation in the original sternal position (Figure 1d,e). Subsequent CT also confirmed the resolution of the abscess and bone fragments as well as new bone formation in the original sternal location (Figure 1f). Oral antibiotics were continued for 5 months. The patient was evaluated for immunodeficiency at the previous hospital through immunoglobulin level and lymphocyte subset testing. Aside from a low IgG2 level of 61.2 mg/dL, no other apparent abnormalities were detected. Upon re‐evaluation at our hospital, the IgG2 level had recovered to 111 mg/dL, and it was not actively considered a contributing factor to susceptibility to infection.

Although this case involved both sequestra and abscess formation, we opted for antibiotic therapy without surgical intervention for the reasons mentioned above. A case series of 74 pediatric primary sternal osteomyelitis cases reported that five out of six cases of abscess formation recovered without surgical intervention.^1^ Our approach was further justified by our ability to closely monitor treatment progress through frequent ultrasonography examinations and our readiness to promptly implement surgical treatment if needed.

In this case, the sternal segments detached, subsequently disappeared, and new bone formation was observed in the original sternal location. At birth, the sternum typically has 4–5 ossification centers, from which ossification spreads. By 1 year of age, ossification extends to the lower part of the sternal body, while ossification of the xiphoid process is further delayed, taking over 3 years. Complete bony fusion of the sternal body occurs around age 30.^3^ These characteristics may explain why multiple segments detached in this case. Detailed reports of sternal segment detachment and subsequent new bone formation in patients with sternal osteomyelitis are rare, making this case a valuable contribution to the literature. We were able to closely and repeatedly monitor the progression of sternal osteomyelitis and surrounding structural changes using ultrasound. One key factor enabling such detailed observation was the sternum's anatomical characteristics as a flat bone located near the surface, which facilitated clear imaging. Additionally, given the patient's age and the early stage of ossification, ultrasound proved particularly useful, as progressive ossification can lead to acoustic shadowing, limiting sonographic visibility in older patients.

## AUTHOR CONTRIBUTIONS

All the authors meet the ICMJE authorship criteria. Hiroki Shimono drafted the initial manuscript. Akiko Kinumaki supervised writing the case report and critically reviewed the manuscript. Natsuho Adachi, Hiroyuki Tanaka, Yuko Kajiho, and Shoichiro Kanda critically reviewed and revised the manuscript. All the authors have read and approved the final manuscript.

## CONFLICT OF INTEREST STATEMENT

The authors declare no conflict of interest.

## ETHICS STATEMENT

Informed consent was obtained from the parents of the patient to publish the data in this report.
